# Testing an active intervention to deter researchers’ use of questionable research practices

**DOI:** 10.1186/s41073-019-0085-3

**Published:** 2019-11-29

**Authors:** S. V. Bruton, M. Brown, D. F. Sacco, R. Didlake

**Affiliations:** 1 School of Humanities, The University of Southern Mississippi, 118 College Drive, #5037, Hattiesburg, MS 39406-0001 USA; 2 School of Psychology, Fairleigh Dickinson University, Williams Hall 204A, 1000 River Rd, Teaneck, NJ 07666 USA; 3 School of Psychology, The University of Southern Mississippi, 118 College Drive, #5025, Hattiesburg, MS 39406-0001 USA; 4 Center for Bioethics and Medical Humanities, University of Mississippi Medical Center, 2500 North State Street, Jackson, MS 39216 USA

**Keywords:** Questionable research practices, Psychological intervention, Ethical evaluation

## Abstract

**Introduction:**

In this study, we tested a simple, active “ethical consistency” intervention aimed at reducing researchers’ endorsement of questionable research practices (QRPs).

**Methods:**

We developed a simple, active ethical consistency intervention and tested it against a control using an established QRP survey instrument. Before responding to a survey that asked about attitudes towards each of fifteen QRPs, participants were randomly assigned to either a consistency or control 3–5-min writing task. A total of 201 participants completed the survey: 121 participants were recruited from a database of currently funded NSF/NIH scientists, and 80 participants were recruited from a pool of active researchers at a large university medical center in the southeastern US. Narrative responses to the writing prompts were coded and analyzed to assist post hoc interpretation of the quantitative data.

**Results:**

We hypothesized that participants in the consistency condition would find ethically ambiguous QRPs less defensible and would indicate less willingness to engage in them than participants in the control condition. The results showed that the consistency intervention had no significant effect on respondents’ reactions regarding the defensibility of the QRPs or their willingness to engage in them. Exploratory analyses considering the narrative themes of participants’ responses indicated that participants in the control condition expressed lower perceptions of QRP defensibility and willingness.

**Conclusion:**

The results did not support the main hypothesis, and the consistency intervention may have had the unwanted effect of inducing increased rationalization. These results may partially explain why RCR courses often seem to have little positive effect.

## Introduction

Over the past several years, scientists have become increasingly concerned about the prevalence of questionable research practices (QRPs) in published scientific research. Alternatively referred to as detrimental research practices [1], QRPs are common, often problematic, research behaviors that are typically ethically more ambiguous than data fabrication or falsification but nonetheless adversely impact the scientific literature (for a more rigorous definition, see [2]). In some cases, QRPs cause harm tangibly and directly, such as when they affect prescribed medical care or waste research funds. In other instances, the harms can be more diffuse, such as when they lead to irreproducible findings, when they delay or prevent the discovery of misconduct and the refutation of mistaken results, and when they contribute to poor student training [1]. One recent analysis estimates $28 billion a year in preclinical biomedical research alone in the USA is wasted on “research that cannot be replicated” ([3]; see also [4]). Despite the fact that they typically fall short of outright fraud, their negative repercussions can be equally lasting and profound [5, 6].

Although no definitive list exists of QRPs, typically cited examples include so-called *p*-hacking (“significance chasing,” or “HARKing”), publication bias, selective citation, reporting of underpowered studies, presentational “spin,” salami-slicing, inappropriate authorship designations, and several others. While surveys show that the percentage of scientists who admit to outright data fabrication or falsification is quite low—somewhere in the range of 1–2% [5]—QRP use appears to be much more common. In some studies, up to a third of scientists acknowledge using some kinds of QRPs, such as changing methodology or results to please a funding source [5]. One well-known study reported that a majority of psychologists had engaged in dubious behavior such as failing to report all dependent measures and excluding data post hoc [7]. While the prevalence of QRPs in the fields of psychology and medicine have received the most scrutiny [8–10], evidence of pervasive QRP use is emerging in disciplines as otherwise disparate as ecology, evolutionary biology, economics, communication science, and environmental toxicology [11–14]. A prominent narrative, frequently encountered in both scientific publications and in the popular press, is that science is in crisis, beset by widespread problems of bias and lack of reproducibility and replication, problems that are at least partly attributable to the prevalence of QRPs. In a 2016 survey of 1500 scientists, 52% of respondents agreed with this conclusion [15].

Whether or not science is in “crisis,” it would clearly be desirable to find effective means of mitigating QRP use. Improved training and education are often advocated in this regard [16], but research ethics-specific training has been shown to have minimal lasting impact [17, 18], despite sustained efforts to improve it [19]. Moreover, reforming current practices will be a gradual process at best. For many norms of practice, it is not entirely clear how best to improve them, and in any case, scientists are often resistant to change, regardless of how well-conceived the changes may be. A recent study of psychologists found surprisingly high levels of failure to adopt some of the most commonly advocated reforms with a predictable variety of rationalizations for these failures [20]. Moreover, many of science’s more stringent means of norm enforcement—peer review, federal and institutional oversight, and formal sanctions—are ill-suited for practices that are often ethically ambiguous and highly dependent on professional judgment. Whether, for example, “changing study design to please a funding source” constitutes something ethically dubious or ethically benign may be difficult to assess without significant additional context and expertise specific to the discipline and project in question. Similarly, the kinds of behavior called QRPs often defy exact specification. Consider that the recent US National Academy of Sciences report lists “misleading” statistical analysis, which falls short of falsification as a detrimental practice [1], but what counts as “misleading” is hard to define precisely. Also, regulatory and enforcement-based approaches tend to foster a “don’t get caught” attitude [21], which is importantly different than the dispositional moral integrity needed for sound science.

While the problem of QRP use resists a simple and quick fix, it might be partially lessened if a direct psychological means of encouraging research integrity could be found. The present study aimed to do just that; we used recent findings from the empirical ethics literature to design a simple intervention geared towards reducing researchers’ acceptance of QRPs. Researchers have long known that individuals’ ethical decision-making is often skewed by motivated reasoning [22, 23]. Rather than rely solely on sound moral principles, people typically make decisions more egoistically, shading the meaning of the relevant principles to their present advantage [24]. Reasoning in scientific contexts is particularly susceptible to unconscious distortions, given that the interpretation of data, relative to available hypotheses and explanations, is subtle and psychologically complex, readily vulnerable to confirmation bias and other self-serving tendencies [25, 26]. Indeed, people with the cognitive sophistication required for serious science are particularly prone to have difficulty recognizing their own biases, a phenomenon known as the “bias blind spot” [27, 28]. Moreover, the increasingly collaborative nature of science can exacerbate cognitive failings, as it facilitates diffusion of responsibility [29, 30]. Though no empirical evidence establishes the link directly, it is plausible to think that QRP use may be in part the result of various kinds of such motivated reasoning. Given the reality of a very tight job market, a highly competitive funding environment [31], ever-increasing emphasis on the quantity of one’s publications as a gauge of professional merit, and the oft-cited “publication bias” towards novel, positive findings [32], it is reasonable to believe that science is negatively affected by both the unintentional and intentional use of QRPs.

The strategy behind our intervention was to use researchers’ presumed motivation for a positive moral self-concept coupled with humans’ well-established desire to avoid cognitive dissonance to induce negative affect towards QRPs. One basis for this approach was that in prior research using the same list of QRPs and “passive” interventions, the consistency intervention showed the most promise, particularly among early-career researchers [33]. Another basis was that in various contexts, activating individuals’ self-concepts and their basic moral commitments has been shown to inspire ethical behavior. In an application of objective self-awareness theory [34], one study showed that signing one’s name before reporting information (rather than afterward) elicited heightened honesty ([35], see also [30, 36]). Such signing is analogous to the time-honored practice of verbally pledging truthfulness before giving testimony in court; it is a way of activating attention to the self. People are also motivated to remain true to their own norms and identities, and reminding them of their identities motivates integrity [37, 38]. For example, the gesture of putting one’s hand over one’s heart has been shown to reduce cheating and encourage honesty [39], similar to the “pro-truth pledge,” an effort currently being tested to combat the effects of “fake news” [40]. Pre-commitment to moral values has been shown an effective means of positively influencing behavior in a range of circumstances, such as by increasing participation in public elections [41] and recycling programs [42].

A short, summary version of the findings below was presented at the 6th World Conference on Research Integrity in Hong Kong [43].

## Methods

An active intervention aimed at reducing researchers’ endorsement of QRPs was tested against a control. Participants in the intervention condition were hypothesized to indicate less approval for QRPs and less willingness to engage in them as compared to control. Prior to beginning data collection, the project was approved by Institutional Review Boards both at the University of Southern Mississippi (Approval #CH2-17102605) and at the University of Mississippi Medical Center (Approval #2018-0069) and pre-registered at Open Science Framework (“Testing Active Interventions to Reduce Questionable Research Practices,” at https://osf.io/uspek/) where all research materials and data are available (Additional file 6).

Additionally, we formulated ancillary hypotheses to test the extent to which age [33] and gender [44] are specifically influenced by these interventions, given previous findings suggest similar interventions are especially effective at ameliorating QRP endorsement among early-career scientists [33] and those indicating gender differences in risk-taking [44]. Analyses of these variables are provided in the supplemental materials for the sake of providing a more comprehensive set of analyses with the available data.

### Sample

Participants were solicited via emails sent out in waves to two populations. One sample was drawn from a list of researchers with active NIH/NSF funding. Another sample was comprised of active research scientists at the University of Mississippi Medical Center (UMMC) in Jackson, MS. This multi-pronged approach allowed for more expeditious collection of data. Based on a power analysis to detect medium-sized effects (Cohen’s *d* = 0.4, *β* = 0.80), a total of 200 participants were sought (*N* = 200) in roughly equal numbers from each population. The desired participation was attained by means of 14 waves of invitation emails to 200 prospective participants per wave (2800 total). A total of 201 individuals completed the survey, 121 from the NIH/NSF and 80 from UMMC, with 98 and 103 participants in the consistency and control conditions, respectively. A preliminary statistical analysis considering the source of the data as a factor yielded no significant effects, thus prompting us to collapse across both data sources.

To acquire this sample, we sent out an invitation email to a researcher-generated listserv of prospective respondents in waves of 200–300 researchers over the course of a month. A new wave was sent to additional prospective respondents every two days. All respondents completed the writing task and thoughtfully responded to their respective prompts, prompting us not to exclude any participants from final analyses for noncompliance.

### Materials and procedure

Participants were randomly assigned either to the consistency condition (intervention) or the control condition and were asked to complete a brief (3–5 min) writing task. Participants in the consistency condition were instructed to write about how they model research integrity in their work and how it is consistent with their core ethical standards; participants in the control condition were asked to write about why fabrication, falsification, and plagiarism are ethically objectionable (Table 1). Narrative responses to the writing task were collected and subsequently coded to inform the analysis of quantitative data (see below).

**Table 1:** Writing prompts for 3–5 min writing tasks

Consistency	
a) Over the past few years, scientists have become increasingly aware of how various ethically questionable research practices can lead to poor science and reduce the ability of scientific research to improve human understanding and well-being. Please begin by spending 3–5 min writing (in the box below) about how you attempt to model research integrity in your own work and with those you mentor, and how this commitment is consistent with your core ethical standards.	
Control	
b) Research misconduct, standardly defined, consists of falsification, fabrication, and plagiarism (FFP). It can lead to poor science and reduce the ability of scientific research to improve human understanding and well-being. Please begin by spending 3–5 min writing (in the box below) about why falsification, fabrication, and plagiarism are ethically objectionable.	

#### Measures

Participants were then asked to respond to two primary dependent measures assessing endorsement of QRPs through perceptions of their overall defensibility and the extent to which participants would be willing to engage in them. Participants also responded to three secondary dependent measures from a “motives questionnaire” to identify potential mechanisms for such endorsements.

##### Primary measures

Participants indicated the extent to which they endorsed 15 QRPs. Specifically, they were given an array of previously validated QRPs identified as representing ethically ambiguous practices [45], and participants were tasked with indicating the extent to which each QRP was ethically defensible and their willingness to engage in each. They indicated their assessment of the ethical defensibility of each QRP using a 7-point Likert-type scale (1 = *completely indefensible*; 7 = *completely defensible*) and the extent to which they would be willing to engage in the described behavior (1 = *completely unwilling to engage in this behavior*; 7 = *completely willing to engage in this behavior*). Each scale was an aggregation of the 15 items, with higher scores indicating greater endorsement of QRPs. The items demonstrated acceptable reliability, suggesting that participants were responding to each item similarly as in previously validated studies (Cronbach’s *α*s > 0.80) [45].

##### Secondary measures

The motives questionnaire asked participants about the impact on others of engaging in the QRPs (3 items; 1 = *very small*; 7 = *very large*), why they might engage in QRPs were they to do so (3 items; 1 = *strongly disagree*; 7 = *strongly agree*), and the potential risks of using QRPs (6 items; 1 = strongly disagree; 7 = strongly agree). Respectively, higher scores reflected perceptions of greater impact of QRPs, greater rationalization of such behaviors, and perceptions of more risk related to QRP use. As with the primary measure, items were aggregated into single-score responses, all of which had acceptable reliabilities (Cronbach’s *α*s > 0.75).

Consenting participants were initially randomly assigned to one of the two interventions through an online randomizing feature in Qualtrics that precludes researchers from actively assigning participants to a condition, thereby reducing potential experimenter bias. Participants then responded to the primary measures and secondary measures. Following completion of the survey questions, participants provided demographic information and were debriefed with the option given of supplying an email address to be sent a $10 Amazon gift card code. Email addresses were automatically de-linked from survey responses in Qualtrics (see Additional files 1, 2, and 3 for research materials).

### Analysis

#### Primary analysis

To identify the basic efficacy of the intervention, we conducted five independent-samples *t* tests to compare participants’ responses between the consistency condition and the control condition. We computed effect sizes, confidence intervals, and mean differences for each analysis. (Secondary analyses of participant responses and demographics were also conducted and can be found in Additional file 4.)

#### Narrative analysis

Given the possibility that narrative themes could serve as proxies for participants’ intentions and therefore predict behavioral motivations, we conducted a series of exploratory analyses based on the narrative responses to the writing prompts. In particular, we were interested in determining how specific ethical concerns mentioned in the narratives might predict responses to QRPs across our dependent measures.

Our initial step was to identify recurring themes in participants’ responses. We first reviewed participant narratives with the goal of identifying fine-grained thematic content ([46]; see also [47]). After exploring the possible interrelatedness of sub-themes, the first two authors successively coded the first 50 responses and then the first 100 responses, comparing results after each attempt and discussing discrepancies. Ultimately, we arrived at four distinct subordinate themes amenable to quantitative thematic analysis: (1) concern for other individuals, including risks to medical patients or research participants and the harm of coercion (respondent with this theme present, *n* = 72); (2) concern for scientific integrity and the search for the truth, such as a focus reproducibility, replicability, transparency, sound statistical analysis, and research design (*n* = 136); (3) concern for broader ethical values and personal virtues, such as an emphasis on personal integrity or accountability (*n* = 69); (4) concern for good mentoring and training (*n* = 45; see Additional file 5 for sample participant responses grouped by coded themes).

The complete set of narrative responses were then coded independently by the first and second author, identifying the presence of each theme in the narratives by coding the theme’s presence as a “1” and absence as a “0.” Using Cohen’s kappa as a gauge of inter-rater reliability, we found an acceptable reliability between coders (*κ*s > 0.79). Because no interactive effects emerged for Themes 2 and 3, we considered them no further. Below we report interactive effects that emerged for Themes 1 and 4.

## Results

### Primary measures

#### Defensibility

No significant difference emerged between the consistency (*M* = 3.01, *SD* = 0.85) and control conditions (*M* = 2.94, *SD* = 0.83) in perceptions of QRPs as defensible, *t*(199) = 0.96, *p* = 0.546, *d* = 0.08, 95% CI [− 0.16, 0.30], *M*_Diff_ = 0.07.

#### Willingness

No difference emerged between the consistency (*M* = 2.83, *SD* = 0.93) and control conditions (*M* = 2.63, *SD* = 0.94) in willingness to engage in QRPs, *t*(198) = 1.50, *p* = 0.134, *d* = 0.21, 95% CI [− 0.06, 0.46], *M*_Diff_ = 0.20.

### Secondary measures

#### Impact

Participants in the consistency (*M* = 3.91, *SD* = 1.79) and control conditions (*M* = 4.18, *SD* = 1.77) did not differ in perceptions of QRPs as impactful, *t*(194) = − 1.04, *p* = 0.300, *d* = 0.15, 95% CI [− 0.76, 0.23], *M*_Diff_ = − 0.26.

#### Risk

No difference emerged in perceptions of QRPs as risky between the consistency (*M* = 5.45, *SD* = 1.52) and control conditions (*M* = 5.74, *SD* = 1.37), *t*(199) = − 1.49, *p* = 0.138, *d* = 0.21, 95% CI [− 0.68, 0.09], *M*_Diff_ = − 0.29.

#### Rationalization

No difference emerged in the consistency (*M* = 2.71, *SD* = 1.56) and control conditions (*M* = 2.34, *SD* = 1.29) in the rationalization of QRPs, *t*(188.11) = 1.79, *p* = 0.074, *d* = 0.25, 95% CI [− 0.03, 0.77], *M*_Diff_ = 0.36.

### Exploratory narrative analyses

We conducted exploratory 2 (condition: consistency vs. control) × 2 (theme: presence vs. absence) factorial ANOVAs for our outcome measures to identify potential effects of themes in influencing participants as a function of the condition to which they were assigned. Given that we sought to reduce the Type I Error rate from reporting the condition effects a second time and that we had largely heterogeneous samples for the presence of each time, we considered the interactive effects in these analyses exclusively. No significant interactive effects emerged for Themes 2 and 3, and we therefore do not report those findings here.

#### Defensibility

Effects were qualified by a 2-way interaction, *F*(1, 197) = 5.10, *p* = 0.025, *η*^*2*^_*p*_ = 0.025. Among participants who wrote about Theme 1, simple effects tests indicated that consistency-primed participants reported greater defensibility of QRPs (*M* = 3.08, *SD* = 0.80) than control participants (*M* = 2.66, *SD* = 0.87), *F*(1, 197) = 4.56, *p* = 0.034, *η*^*2*^_*p*_ = 0.023, 95% CI [0.03, 0.81], *M*_Diff_ = 0.42. However, the absence of this theme resulted in no difference in defensibility among consistency-primed (M = 2.97, SD = 0.87) and control participants (*M* = 3.11, *SD* = 0.77), *F*(1, 197) = 0.83, *p* = 0.363, *η*^*2*^_*p*_ = 0.004, 95% CI [− 0.42, 0.15], *M*_Diff_ = 0.13. Viewed another way, consistency-primed participants did not differ in perceived defensibility based on the presence of Theme 1, *F*(1, 197) = 7.07, *p* = 0.008, *η*^*2*^_*p*_ = 0.035, 95% CI [0.11, 0.78], *M*_Diff_ = 0.45, whereas control participants reporting Theme 1 perceived QRPs as less defensible than control participants not writing about Theme 1, *F*(1, 197) = 0.34, *p* = 0.557, *η*^*2*^_*p*_ = 0.002, 95% CI [− 0.45, 0.24], *M*_Diff_ = 0.10.

#### Willingness

Effects were qualified by a 2-way interaction, *F*(1, 196) = 7.20, *p* = 0.008, *η*^*2*^_*p*_ = 0.035. Among participants who wrote about Theme 1, simple effects indicated that consistency-primed participants were more willing to engage in QRPs (*M* = 2.86, *SD* = 0.92) than control participants, (*M* = 2.21, *SD* = 0.89), *F*(1, 196) = 9.02, *p* = 0.003, *η*^*2*^_*p*_ = 0.004, 95% CI [0.22, 1.08], *M*_Diff_ = 0.65. However, the absence of this theme resulted in no difference between consistency-primed (*M* = 2.81, *SD* = 0.94) and control participants (*M* = 2.89, *SD* = 0.89), *F*(1, 196) = 0.21, *p* = 0.644, *η*^*2*^_*p*_ = 0.001, 95% CI [− 0.39, 0.24], *M*_Diff_ = 0.07. Viewed another way, no difference emerged in willingness for consistency-primed participants as a function of Theme 1 presence, *F*(1, 196) = 0.06, *p* = 0.795, *η*^*2*^_*p*_ < 0.001, 95% CI [0.30, 1.04], *M*_Diff_ = 0.67, whereas control participants reported less willingness to engage in QRPs when Theme 1 was present than if it was absent, *F*(1, 196) = 13.08, *p* < 0.001, *η*^*2*^_*p*_ = 0.063, 95% [− 0.43, 0.33], *M*_Diff_ = 0.05.

#### Risk

Effects were qualified by a 2-way interaction, *F*(1, 197) = 5.28, *p* = 0.023, *η*^*2*^_*p*_ = 0.026. Among participants who wrote about Theme 1, simple effects indicated that control participants perceived QRPs as riskier (*M* = 6.10, *SD* = 0.92) than did consistency-primed participants (*M* = 5.21, *SD* = 1.52), *F*(1, 197) = 7.43, *p* = 0.007, *η*^*2*^_*p*_ = 0.036, 95% CI [0.24, 1.54], *M*_Diff_ = 0.89. Conversely, no difference emerged between control (*M* = 5.53, *SD* = 1.55) and consistency-primed participants (*M* = 5.58, *SD* = 1.35) when Theme 1 was absent, *F*(1, 197) = 0.03, *p* = 0.850, *η*^*2*^_*p*_ < 0.001, 95% CI [− 0.43, 0.52], *M*_Diff_ = 0.04. Viewed another way, the presence of Theme 1 elicited perceptions of QRPs as riskier among control participants compared to its absence, *F*(1, 197) = 4.09, *p* = 0.044, *η*^*2*^_*p*_ = 0.020, 95% CI [− 1.12, − 0.01], *M*_Diff_ = 0.57, whereas no difference emerged between presence and absence among consistency-primed participants, *F*(1, 197) = 1.56, *p* = 0.213, *η*^*2*^_*p*_ = 0.008, 95% CI [− 0.21, 0.95], *M*_Diff_ = 0.36.

Interactions for rationalization and impact were not significant and therefore considered no further.

### Theme 4

We used similarly dimensioned ANOVAs for Theme 4. A 2-way interaction emerged for Impact, *F*(1, 192) = 4.74, *p* = 0.031, *η*^*2*^_*p*_ = 0.024. Among participants who wrote about Theme 4, simple effects indicated that control participants viewed QRPs as more impactful (*M* = 6.00, *SD* = 0.88) than consistency-primed participants (*M* = 3.62, *SD* = 1.50), *F*(1, 192) = 5.06, *p* = 0.026, *η*^*2*^_*p*_ = 0.026, 95% CI [0.29, 4.45], *M*_Diff_ = 2.37. However, among participants for which Theme 4 was absent, no difference emerged between consistency-primed (*M* = 4.14, *SD* = 1.96) and control participants (*M* = 4.12, *SD* = 1.76), *F*(1, 192) < 0.01, *p* = 0.962, *η*^*2*^_*p*_ < 0.001, 95% CI [− 0.57, 0.60], *M*_Diff_ = 0.01. Viewed another way, no differences emerged in impact when comparing presence and absence of Theme 4 between conditions, *F*s < 3.30, *p*s > 0.07.

No other interactions emerged among the other measures, prompting us to consider them no further, *F*s < 3.22, *p*s > 0.07.

## Discussion

Overall, the main research hypothesis was not supported. The consistency intervention did not reduce perceptions of the defensibility of QRPs or willingness to engage in them relative to control. A possible explanation for this result is that both the consistency and control writing tasks produced similar responses, because both tasks involved reflection on ethical norms, participants responded with similar reactions to QRPs. However, this explanation does little to explain the most salient effect of the intervention, the inducement of a greater tendency to rationalize use of QRPs (supposing one were to use them), particularly by women.

A possible explanation for this increased rationalization is that by priming researchers’ thoughts of themselves as morally conscientious, participants were encouraged to regard problematic potential future behaviors as reasonable. Research on dishonesty in a variety of contexts has shown that most people will cut ethical corners to their own advantage on the condition that they can do so without undermining their positive self-concept [36, 48]. Most people will lie and cheat at least a little, but only insofar as their self-image is maintained. Buttressing researchers’ ethical self-concepts via the consistency intervention may have helped participants excuse prospective questionable behavior without changing their disposition towards engaging in it.

The psychology literature suggests several different ways self-concept maintenance may have been triggered by the consistency intervention. One possibility, the phenomenon of moral licensing, is the process whereby individuals display a tendency to behave less ethically after recent displays of rectitude. Such licensing has been demonstrated as an unintended negative effect of financial conflict of interest disclosure [49], and it is consistent with rather surprising findings from a research ethics context. Specifically, students who had received research ethics training were subsequently less willing to take moral responsibility for their actions [18]. A similar directionality of effect can be seen in studies on moral distancing. When faced with explaining questionable behavior, individuals often seek to distance themselves from it by blaming the behavior on external forces, claiming “everybody does it,” and the like [50, 51]. In essence, with heightened activation of their own moral commitments, participants in the consistency condition felt a greater need to deny their agency regarding possible future misdeeds. An analogous tendency has also been documented in studies on moral hypocrisy. In certain experimental contexts, participants induced to have a greater sense of their own moral responsibility display not greater integrity, as one might expect, but evidence of greater hypocrisy [52, 53]. In such circumstances, activating individuals’ sense of their own moral conscientiousness affects their self-presentation but does not ameliorate their questionable behavior. Rationalization is a kind of self-presentation.

These suggestions also seem to cohere with the interesting relationships that emerged between our quantitative findings and narrative coding for Themes 1 and 4. Both themes indicate activation of thoughts about the way other people may be adversely and directly affected by QRPs. Whether thinking of one’s students and trainees, as in the case of Theme 4, or medical patients in one’s care, as in the case of Theme 1, the focus is on specific individuals, as opposed to more generalized concerns of scientific ethics such as experimental replicability, or scientific truth, and the like. Consequently, one might expect participants whose responses coded for Themes 1 and 4 to express somewhat less support for QRPs than those participants whose responses did not reveal these themes. Indeed, participants who coded for Theme 1 in the control condition found QRPs riskier and less defensible and they expressed less willingness to engage in them. However, no such tendency emerged for participants in the consistency intervention, the condition which also increased rationalization. Similarly, the responses of participants in the control condition that coded for Theme 4 also regarded QRPs as having greater adverse impact, but no such relationship held for participants in the consistency condition. As with our quantitative results, the consistency intervention seemed to mitigate the extent to which participants who were mindful of how their work influenced others perceived QPRs more negatively.

### Limitations

Various possibilities could explain why the current intervention had such limited efficacy. As is true of other survey-based studies about ethics, legitimate questions can always be raised about possible discrepancies between actual behavior and responses to hypothetical actions [54]. Within the context of this study, this discrepancy could be highlighted by a bias towards socially desirable responding, given the deleterious consequences of engaging in QRPs [55]. That is, participants may have self-censored their responses to some degree, which could help explain reductions in reported QRPs. Recent interventions have covaried out respondents’ proclivity towards socially desirable responding to account for this self-censorship, thereby necessitating measuring this tendency in subsequent studies [56]. Future research would benefit from tasking participants with communicating their endorsement of QRPs in less direct ways, further removed from their own possible culpability. For example, a future study could put participants in the role of serving as peer reviewers for a journal manuscript that manifested evidence of QRPs. Participants might then assess the extent to which they would request clarification of relevant details before recommending publication.

Another possible limitation of the current study involves the immersion of participants in the writing prompt. Although previous findings suggest that approximately 5 min of writing is sufficient to elicit a desired motivational state [57, 58], that time may not suffice for the self-relevant information at issue here. Future research would benefit from considering more intensive immersion primes that might elicit self-other consistency more effectively [59].

Another possible limitation pertains to the consistency intervention itself. While prior research suggested its promise, it may well be that prompting different thoughts might have worked much better. For example, it might be that an opposite strategy, one of completely de-emphasizing the role of the self, might have been more effective. A prompt encouraging participants to think about the long-term effects of QRPs and their impact on other researchers and fellow citizens might have greater impact and might not promote the kind of rationalizing response evidenced here. This non-egocentric perspective is perhaps easier for later-career researchers to achieve, given the tendency of their developmentally appropriate concerns to be more focused on others [60].

Although our analyses with narratives were exploratory, used as a means to develop a better understanding of the intervention for future research, we must nonetheless urge caution in the interpretation of findings from the narrative analyses. The overall sample size for the subgroup analyses in the reported analyses could be the result of limited power, particularly as regards Theme 4. Indeed, Theme 1 likely had a sufficient number of respondents with the themes being absent or present, based on sampling in previous research investigating narrative differences [46], but future research is ultimately necessary to determine how robust these findings are a priori.

Previous research has additionally indicated that overall experience in one’s field is especially predictive of engagement in QRPs. That is, early-career researchers appear more prone to QRP endorsement when not focused on consistency between their research identities and research ideals [45]. Perhaps early-career researchers are aggressive in a way that gives them a greater propensity for marginally ethics behavior. This possibility too warrants exploration in future research.

## Conclusion

While lack of support for the main research hypothesis is seemingly at odds with some of the findings from the social science literature used as a basis for the intervention, it is consistent with studies that show limited effectiveness of RCR education. While considerable effort has been expended over the past three decades to develop effective RCR training materials and methods, evidence on their impact is equivocal [17, 61]. Some studies show marginal benefit ([1], Appendix C), others little if any positive impact [62], while some studies show that RCR has unwanted negative effects [18]. These latter findings are consistent with the directionality of the results of the intervention tested here insofar as it appeared to have an unwelcome tendency to induce rationalization. In some contexts, RCR training has been shown to result in trainees’ overconfidence in their ability to handle problems and an overemphasis on their ethicality [18].

Cumulatively, the relative lack of solid evidence in support of RCR training suggests the need for alternative approaches. While most efforts to improve the ethics of scientific practice have focused on reforming individuals’ awareness and compliance, as is true of the technique tested in the present study, perhaps reforms to research environments that are more systematic and institutional are the better approach [63, 64]. Over the long-term, significant advances in scientific practice and adherence to ethical norms may require policy-based measures, social and cultural reforms, and altered institutional structures [65]. To a certain extent, changes in this direction are already underway, such as increasing expectations to pre-register study methods and hypotheses, efforts to enhance transparency and access to experimental data, stricter oversight by journals, and so forth. But more can and should be done in these veins, and to date, reforms have not been adopted as widely or as systematically as would be optimal.

## Supplementary information

**Additional file 1.** QRP Endorsement Questionnaire.

**Additional file 2.** Motives Questionnaire.

**Additional file 3.** Demographics Questionnaire.

**Additional file 4.** Secondary Analyses.

**Additional file 5.** Narrative Coding Examples.

**Additional file 6.** Active Interventions Study Protocol.

## Data Availability

This study was pre-registered as “Testing Active Interventions to Reduce Questionable Research Practices,” at the Open Science Framework, at https://osf.io/uspek/. Research materials and data are available from this site.

## Abbreviations

QRP: Questionable research practice
FFP: Fabrication, falsification, and plagiarism
