# Supplementary material for: Testing an active intervention to deter researchers’ use of questionable research practices
Source: Res Integr Peer Rev. 2019 Nov 29;4:24. doi: 10.1186/s41073-019-0085-3 (PMC6883712; doi:10.1186/s41073-019-0085-3)
Supplement: Supplementary file 1 — Additional file 1. QRP Endorsement Questionnaire. [file 41073_2019_85_MOESM1_ESM.docx]

**S1 QRP Endorsement Questionnaire**

To what extent is this behavior ethically defensible?

| Completely Indefensible | Moderately Indefensible | Somewhat Indefensible | Neither Defensible nor Indefensible | Somewhat Defensible | Moderately Defensible | Completely Defensible |
| --- | --- | --- | --- | --- | --- | --- |
| 1 | 2 | 3 | 4 | 5 | 6 | 7 |

To what extent would you be willing to engage in this behavior?

| Completely Unwilling to Engage in this Behavior | Moderately Unwilling to Engage in this Behavior | Somewhat Unwilling to Engage in this Behavior | Neither Willing nor Unwilling to Engage in this Behavior | Somewhat Willing to Engage in this Behavior | Moderately Willing to Engage in this Behavior | Completely Willing to Engage in this Behavior |
| --- | --- | --- | --- | --- | --- | --- |
| 1 | 2 | 3 | 4 | 5 | 6 | 7 |

**QRPs**

1. To enhance chances of publication, violating the ideal of “replace, reduce, refine” regarding the use of research animals.

2. Adding additional research participants because the results collected thus far are not yet statistically significant.

3. Stopping collecting data earlier than planned because the hypothesized result already had been attained.

4. Rounding off a *p* value simply to make results seem more significant, such as by reporting a *p* value of .044 to be *p* = .04.

5. Deciding whether to include or exclude data after looking at the impact of doing so on the results.

6. Failing to report all of a study’s outcome measures.

7. Selectively discussing only studies that supported the hypothesized result(s).

8. Reporting an unexpected result as having been hypothesized from the start.

9. Drawing strong inferences from statistically significant but underpowered results.

10. Selective reporting of subgroups, outcomes, and time points.

11. Deliberately delaying reporting results in order to publish findings in a higher impact journal.

12. Re-use of one’s own previously published ideas or words without citation, such as parts of a literature review section, introduction or methodology, but without re-using data, results, or analysis.

13. Publishing results of a single study as several articles simply to increase the number of publications derived from the research (the so-called “salami slicing” problem).

14. Changing the design, methodology or results of a study to please a sponsor.

15. Acknowledging another’s technical assistance in publication without that person’s permission.
