# Supplementary material for: Testing an active intervention to deter researchers’ use of questionable research practices
Source: Res Integr Peer Rev. 2019 Nov 29;4:24. doi: 10.1186/s41073-019-0085-3 (PMC6883712; doi:10.1186/s41073-019-0085-3)
Supplement: Supplementary file 2 — Additional file 2. Motives Questionnaire. [file 41073_2019_85_MOESM2_ESM.docx]

**S2 Motives Questionnaire**

| Reliability (Established in Previous work)^1^ | | | |
| --- | --- | --- | --- |
|  | Impact | Rationalization | Risk |
| Study 1 | α=.90 | α=.78 | α=.87 |
| Study 2 | α=.93 | α=.77 | α=.85 |

**Impact**

In general, if you were to engage in any of the research behaviors previously described:

| Very Small |  |  |  |  |  | Very Large |
| --- | --- | --- | --- | --- | --- | --- |
| 1 | 2 | 3 | 4 | 5 | 6 | 7 |

What would be the magnitude of the impact on science?

What would be the magnitude of the impact on society?

How large would the impact be on other individuals?

**Rationalization**

If you were to engage in any of the research behaviors previously described, why might you do so?

| Strongly Disagree |  |  |  |  |  | Strongly Agree |
| --- | --- | --- | --- | --- | --- | --- |
| 1 | 2 | 3 | 4 | 5 | 6 | 7 |

It would not be a big deal because no one would be directly hurt or affected.

I would only be doing what other researchers do all the time.

I would only be responding to systematic career incentives beyond my control.

**Risk**

What might prevent you from engaging in any of the research behaviors previously described?

| Strongly Disagree |  |  |  |  |  | Strongly Agree |
| --- | --- | --- | --- | --- | --- | --- |
| 1 | 2 | 3 | 4 | 5 | 6 | 7 |

They might interfere with my ability to secure extramural funding.

They might damage my professional reputation.

They might damage my reputation within my institution.

They might damage my professional relationships with colleagues.

They might delay or interfere with publication.

They are inconsistent with my own sense of research integrity.

^1^ Sacco DF, Bruton SV, Brown M. In defense of the questionable: defining the basis of research scientists’ engagement in questionable research practices. J Empir Res Hum Res Ethics. 2018;13(1):101-10.
