# Supplementary material for: Testing an active intervention to deter researchers’ use of questionable research practices
Source: Res Integr Peer Rev. 2019 Nov 29;4:24. doi: 10.1186/s41073-019-0085-3 (PMC6883712; doi:10.1186/s41073-019-0085-3)
Supplement: Supplementary file 3 — Additional file 3. Demographics Questionnaire. [file 41073_2019_85_MOESM3_ESM.docx]

**S3 Demographics Questionnaire**

1. What is your age (in years)?

_________

2. What is your gender?

Male
Female
Other

3. Are you Hispanic or Latino (A person of Cuban, Mexican, Puerto Rican, South or Central American, or other Spanish culture or origin)?

Yes
No

4. How would you describe yourself? (Choose one or more of the following)

American Indian or Alaskan Native
Asian
Black or African American
Native Hawaiian or other Pacific Islander
White

5. What is your academic research or academic discipline or field?

______________

6. What is your highest academic degree?

BA
MA
MS
MD
PhD
JD
Other ___________

7. For how many years have you been conducting research in your discipline or field?

_____________

8. How many extramural grants have you been awarded (e.g., National Institutes of Health, National Science Foundation, etc.) in your career?

_____________

9. Approximately how much money (in US dollars) have you been awarded in extramural funding over your career?

_____________
