# Supplementary material for: Testing an active intervention to deter researchers’ use of questionable research practices
Source: Res Integr Peer Rev. 2019 Nov 29;4:24. doi: 10.1186/s41073-019-0085-3 (PMC6883712; doi:10.1186/s41073-019-0085-3)
Supplement: Supplementary file 4 — Additional file 4. Secondary Analyses. [file 41073_2019_85_MOESM4_ESM.docx]

**S4 Secondary Analyses Using Age and Experience**

**Analysis**

The omnibus statistical model was a 2 (Condition: Consistency Motives vs. Control) × 2 (Participant Gender: Male, Female) custom ANCOVA, with participants’ years in their respective fields as a continuous covariate. The custom model feature in SPSS allowed building interaction terms between categorical and continuous predictors along with testing main effect predictions. Significant statistical interactions between Condition and Participant Sex or Condition and Experience were analyzed, with the former decomposed using post hoc t-tests and the latter using bivariate correlations. Effect sizes were computed for all analyses.

# Results

## Defensibility

A statistically non-significant, albeit directionally consistent, main effect of gender emerged, such that men found QRPs more defensible (*M*=2.98, *SD*=0.83) than did women (*M*=2.93, *SD*=0.87), *F*(1, 187)=3.40, *p*=0.067, *η²_p_*=0.018. Effects were further qualified by a trending Gender × Experience interaction, *F*(1, 187)=3.23, p=0.074, *η²_p_* =0.017. An exploratory decomposition of this interaction indicated that men’s increasing years in the field reduced their endorsement of QRPs, albeit non-significantly, *r*(111)=-0.109, *p*=0.252. Women’s increasing years in the field increased their endorsement, also non-significantly, *r*(84)=0.109, *p*=0.320. No other main effects or interactions approached significance, *F*s<0.90, *p*s>0.349. Thus, experience in our sample seemed to have opposing effects on men and women, with men’s QRP defensibility decreasing with experience and women’s increasing.

## Willingness

A significant main effect of gender indicated that men were more willing to engage in QRPs (*M*=2.78, *SD*=0.93) than were women (*M*=2.63, *SD*=0.97), *F*(1, 187)=4.73, *p*=0.031, η²=0.025. Effects were further qualified by a trending Gender × Years interaction, *F*(1, 187)=2.93, *p*=0.088, *η²_p_* =0.015. An exploratory decomposition of this interaction indicated that men’s increasing years in the field reduced their willingness to engage in QRPs, albeit non-significantly, *r*(110)=-0.151, *p*=0.113. No relation emerged for women, *r*(86)=0.093, *p*=0.396. No other main effects or interactions emerged, *F*s<2.28, *p*s>0.136.

## Impact

No main effect or interactions emerged for impact, *F*s<1.82, *p*s>0.179.

## Rationalization

A significant main effect of condition emerged, such that participants in the consistency bias condition rationalized QRPs (*M*=2.69, *SD*=1.56) more than those in the control condition (*M*=2.36, *SD*=1.30), *F*(1, 187)=6.64, *p*=0.011, *η²_p_*=0.034. Another main effect of gender indicated that men (*M*=2.45, *SD*=1.35) rationalized QRPs less than women (*M*=2.63, *SD*=1.55), *F*(1, 187)=5.49, *p*=0.020, *η²_p_*=0.029. Effects were qualified by a Gender × Years interaction, *F*(1, 187)=7.36, *p*=0.007, *η²_p_*=0.038. Decomposition of this interaction revealed that men’s increasing years in the field reduced their rationalization of QRPs, *r*(109)=-0.323, *p*<0.001. No correlation emerged for women, *r*(84)=0.038, *p*=0.728. Effects were further qualified by a trending Condition × Years interaction, *F*(1, 187)=3.23, *p*=0.074, *η²_p_* =0.017. Exploratory decomposition of this interaction indicated that increasing years in the consistency bias condition reduced rationalization of QRPs, *r*(96)=-0.269, *p*=0.007. No correlation emerged for the control condition, *r*(97)=-0.037, *p*=0.713. No other interactions emerged in this analysis, *F*s<0.06, *p*s>0.799.

## Risk

A significant main effect of gender indicated that men found QRPs riskier (*M*=5.67, *SD*=1.25) than did women (*M*=5.53, *SD*=1.52), *F*(1, 187)=5.25, *p*=0.023, *η²_p_*=0.027. Effects were qualified by a trending Gender × Condition interaction, *F*(1, 187)=3.39, *p*=0.067, *η²_p_* =0.018. In the consistency bias condition, per our exploratory analysis, men perceived more risk in QRPs (*M*=5.73, *SD*=1.08) than did women (*M*=5.13, *SD*=1.67), *t*(75.47)=2.07, *p*=0.042. No effect emerged for participants in the control condition, *t*(101)=-1.64, *p*=0.104. Effects were further qualified by a significant Gender × Years interaction, *F*(1, 187)=10.73, *p*=0.001, *η²_p_* =0.054. Decomposition of this interaction indicated that increasing years in the field reduced women’s perceived risk of QRPs, *r*(84)=-0.350, *p*<0.001. No effect emerged for men, *r*(111)=-0.016, *p*=0.863. No other interactions emerged, *F*s<0.46, *p*s>0.503.
