# Supplementary material for: Testing an active intervention to deter researchers’ use of questionable research practices
Source: Res Integr Peer Rev. 2019 Nov 29;4:24. doi: 10.1186/s41073-019-0085-3 (PMC6883712; doi:10.1186/s41073-019-0085-3)
Supplement: Supplementary file 5 — Additional file 5. Narrative Coding Examples. [file 41073_2019_85_MOESM5_ESM.docx]

**S5 Narrative Coding Examples**

Coding Themes for Narrative Responses Analysis with sample responses to the 3 – 5 minute writing prompts. Participants numbered 1 – 98 below were in the consistency condition; participants numbered 99 – 201 below were in the control condition. (Statements below reflect a small number of minor corrections to misspellings and typographical errors.)

| Theme: | Participant Number: | Comment: |
| --- | --- | --- |
| 1. Concern for others | 1.  56.  88.  106.  143.  176. | “Ethical research takes account of the fact that coercion takes many forms and extends to the interpersonal context in which participation in research is offered.”  “Another area in which I still need to improve is the ethics of giving back to participants when it comes to my data. Participants often receive benefits from taking part in the research, but one of those benefits should be a full report of the research findings once they are complete. It is unethical to just “use” people, organizations, etc. I need to do better with this.”  “Important to me is transparency with research participants. First, try to explain as fully as possible in understandable language what the study is about – its major purpose and the kinds of questions they will encounter. Second, let them raise questions and discuss any issues…. Third, answer their questions and get their consent for participation. Once the data are collected, by very sure not to share information gathered with others (except other researchers on the team). Third, once analyses are done, do at least something in a format that can be easily shared with research participants.”  “Especially with medical research, [falsification] leads to misinformation and could potentially put the population at risk for injury or even death. Fabrication within research again is inherently dishonest and could lead to significant harm. The intent of research is to discover and share accurate information that can be proven through similar studies, and disseminated for the good of the public.”  “My immediate thought regarding falsification and fabrication was the ‘end result’, our clients/patient. Any deviation (minor or major) from factual and truthful processes of research could result in harm to our clients.”    “Falsification and fabrication of data leads to poor or incorrect science that can at worst lead to direct harm to others. It can also lead to professional advancement at the expense of others. This is particularly true of plagiarism, which is a form of “taking” of others’ work. Ethically, these situations may lead to personal harm and this is why they are ethically objectionable.” |
| 2. Concern for the practices and values of scientific integrity and the search for truth. | 19.  59.  67.  93.  149.  184. | “I have for a long while engaged routinely in using power analyses in order to preempt ‘fishing’ expeditions. I have also for the vast majority of my papers posted the data analytic protocols to the OSF online repository and included a link to the OSF (with the deidentified dataset for reviewers and later us) in my paper. I have prespecified the data analytic procedures to test hypotheses before data analysis and ensure there is collective agreement among the team before running analyses.”  “As an heir of the Enlightenment, I view science and its methods as value-neutral. In this context, I generally abstract away from ethical considerations in the design of research strategies, and allow external referees to consider, at their discretion, the extent to which a proposed research design has ethical shortcomings.”  “We will employ rigorous and transparent experimental designs and utilize robust tools of statistical inference. We also do not receive or have accepted funding from any interest groups, thus maintain us immune from preconceived and/or desirable research outcomes.”  “I have always tried to quantify our results in a non-biased way. We previously did this by blinding most but not all of our analyses. It is hard because it is frequently patently obvious what is a mutant versus wild-type, but at least this allows us to not know which mutants have received which treatment. We previously did very simple stats in excel. More recently we have learned how to do proper power analyses, test distribution, and choose more appropriate tests.”  “Research misconduct is anathema to the scientific process. It is diametrically opposed to what science is. It is reprehensible because it harms the scientific community both by hindering scientific progress and also by casting a negative light on science in a time when science is under attack from politicians and members of the public.”  “The foundation of science is the search for truth about the natural world, including ourselves. Although science does not claim to know the absolute truth “only closer and closer approximations of it” science is based on the idea that there is an absolute truth, and that it is knowable through the scientific method. Any compromise of scientific integrity, and IMO, there are many more subtle forms of compromise than straight-out FFP, undermines the heart of science.” |
| 3. Concern for broader ethical values and personal virtues | 23.  29.  65.  114.  157.  175. | “My core ethical standards of integrity, honesty, and straightforward carrying out and presentation of research come from my Christian faith.”  “By asking myself what is right and what is wrong. If my intuition and gut feeling say it is wrong to do, then I’d never do it. What is right or wrong is usually distinct. Of course there are gray areas in research, but it is almost always possible to know what is right and what is wrong during research conduct. 2) By asking myself, what would my mentors do if they were in my situations. To model someone with great integrity helps in improving integrity. 3) By following research guidelines for research integrity. Guidelines are available, and they help when the worst happens.”  “I have thought a considerable amount about research integrity since in my previous job I ran a series of workshops on the subject. I try to practice what I believe to be sound ethical principles in my research and teaching. Other than a small number of instances of plagiarism among my undergraduate students many years ago, I have not had much occasion to deal with violations of these principles on a personal level.”  “Everyone knows, inherently, that they should not conduct themselves in such an unethical manner. However, that does not seem to curtail the behaviors. I have seen evidence of these behaviors in all levels of my academic scientific career. What is equally, or perhaps even more, concerning is the common practice of looking the other way. This allows people who are willing to commit these kinds of infractions to continue in their careers and to even be very successful in their careers, when in actuality, they should be punished with some type of consequence.”  “Science is based on accurate observation and experimentation, and scientists have an obligation to report results honestly. Falsification and fabrication are ethically objectionable because they are dishonest and can lead to false scientific results. Plagiarism is also dishonest in that it takes credit for the work of others.”  “We need to be able to trust our fellow scientists to provide accurate and truthful interpretations of the research they are conducting. It is also unethical because it unfairly disadvantages someone over others. A person who falsifies or fabricates data can do so in a way that makes their research seem more perfect than others and thereby providing them with increased opportunities for publications, grants, awards, etc.” |
| 4. Concern for good student mentoring and training. | 38.  42.  52.  108.  192.  198. | “I talk with students about the importance of keeping accurate data and that, regardless of what the data show, it needs to be reported. We talk about how no result or an unexpected result often leads to new directions of research rather than that the data is flawed. I do work with invertebrate animals as well as some educational research. My undergraduate students know about both avenues of work. We talk about IACUC protocols and IRB approvals – why they are needed, how we minimize risk to my dung beetles and to those in the educational research arena.”  “In advising students I focus on the development of important and interesting research questions, the decision about selecting the right methodology or methodologies to address the questions, and designing the study so that it has the potential to answer the question… I teach students to use robust statistical techniques, and to design studies with sufficient statistical power.”  “Research Integrity is an integral part in my training of undergraduate and graduate student researchers. I emphasize the many pitfalls that can compromise experimental conclusions, including psychological ones (inherent bias) as well as problems with setting up and interpreting data (understanding technical and biological replicates, understanding basic statistics, recognizing problems with reagents, especially immunological ones, and problems associated with cell lines.”  “If your mentor is using the elements of falsification and fabrication of data, then you, as a future researcher are learning that these practices are acceptable and necessary for successful publication and promotion… overall, I have never knowingly been involved in an FFP incident and have worked very hard to make sure my students understand what FFP means and giving my students the tools needed to think clearly and critically about data and the reporting of data.”  “As a ‘teacher-scholar’ it strikes me as disingenuous to ask my students to uphold ethical research practices without doing so myself.”  “It’s profoundly important to foster a lab culture that rewards truth and honesty…. There’s no right answer to an experiment, the data is the data and in fact I always tell my students that the surprising results are often the most interesting. I do blind students to genotypes etc. wherever possible, and insist on documentation, back-ups of data etc. so that all results can be easily vetted. I also insist on keeping all such records in the lab. I encourage people to be honest if they break something or make a mistake… rather than sweeping these things under the rung. Finally, it is important to lead by example… never encourage anyone to hide the truth or practice dishonestly.” |
