# Supplementary material for: Testing an active intervention to deter researchers’ use of questionable research practices
Source: Res Integr Peer Rev. 2019 Nov 29;4:24. doi: 10.1186/s41073-019-0085-3 (PMC6883712; doi:10.1186/s41073-019-0085-3)
Supplement: Supplementary file 6 — Additional file 6. Active Interventions Study Protocol. [file 41073_2019_85_MOESM6_ESM.docx]

Study Information

1. Title: Testing Active Interventions to Reduce Questionable Research Practices (2018)

2. Authorship: Bruton, S., Sacco, D., Brown, M., Didlake, R. (order TBD)

3. Research Question:

This study will investigate whether research participants who receive an intervention designed to leverage “consistency” motives will indicate less support for various questionable research practices (QRPs) than participants in the control condition. The list of 15 QRPs to be used are a list of ambiguously unethical QRPs derived from previous work (Sacco, Bruton, & Brown, 2018). Regarding each of the 15 QRPs, participants will be asked the extent to which they find the behavior ethically defensible and the extent to which they would be willing to engage in the behavior. Both questions are to be answered using a 7-point Likert scale. Participants will be randomly assigned to one of two conditions on a between-participants basis: consistency motives activation or control condition. Participants will be instructed to engage in a specific writing task in each condition prior to completing the QRP endorsement questionnaire. All materials are appended below.

4. Hypotheses

Our primary hypothesis is that participants who receive the active consistency intervention will be less likely to find the QRPs ethically defensible and to express a willingness to engage in them.

Our secondary hypotheses, as suggested by our own previous findings, is that the impact of the consistency motives intervention relative to control will be more pronounced for 1) researchers with less experience and female researchers, compared to more experienced researchers and men.

We also intend to collect other demographic information, such as academic field/discipline, which we may use for exploratory analyses to inform subsequent research.

5. Sampling Plan

Data will be collected in Qualtrics on two samples of 100 participants each (N=200). This is based on a power analysis to detect medium-sized effects (Cohen’s d=0.4, β=0.80). One sample will consist of active NIH/NSF-funding researchers. Invitations will be sent out in waves, as in past use of this population (Sacco et al., 2018), until 100 participants have been reached. The second sample will consist of 100 active researchers recruited from University of Mississippi Medical Center (UMMC) in Jackson by Dr. Ralph Didlake.

Participants will be given the option of receiving a $10 Amazon e-gift card in return for participation if they supply email addresses. The email addresses will be automatically de-linked from participants’ responses.

Stopping Rule: Total sample size of 200, which we determined to be sufficient through a power analysis. Responses will be checked daily and data collection will terminated in each sample once 100 participants have validly completed the study.

6. Variables

The key experimental manipulation is condition, consisting of the beginning reading prompt and writing task.

The measured outcome variable is the responses to the two questions (defensibility and willingness to engage) for each of the 15 QRPs.

The study is a no blinding experiment, randomizing participants in Qualtrics into either an experimental intervention (consistency) or control.

We will ensure scale reliability by computing Cronbach’s alpha prior to aggregating across QRPs, consistent with our past research. The omnibus statistical model will be a 2 Condition (Consistency Motives, Control) x 2 Participant Gender (Male, Female) custom ANCOVA, with participants’ years in their respective field as a continuous covariate. The custom model feature in SPSS will allow us to build interaction terms between categorical and continuous predictors, along with testing main effect predictions. If predicted statistical interactions between Condition and Participant Sex or Condition and Experience are significant, the former will be decomposed using simple effects analyses and the latter using simple slopes analyses. Additionally, we will compute and report effect sizes and confidence intervals to accompany the reporting of all analyses.

Below is the wording for the 2 instructions, consistency and control.

7. Materials

Consistency Condition Instructions:

Over the past few years, scientists have become increasingly aware of how various ethically questionable research practices can lead to poor science and reduce the ability of scientific research to improve human understanding and well-being.  Please begin by spending 3 – 5 minutes writing (in the box below) about how you attempt to model research integrity in your own work and with those you mentor, and how this commitment is consistent with your core ethical standards.

Control Condition Instructions:

Research misconduct, standardly defined, consists of falsification, fabrication and plagiarism (FFP). It can lead to poor science and reduce the ability of scientific research to improve human understanding and well-being. Please begin by spending 3 - 5 minutes writing (in the box below) about why falsification, fabrication and plagiarism are ethically objectionable.

QRP Questionnaire:

For each of the Questionable Research Practices below, participants will be asked:

To what extent is this behavior ethically defensible?

| Completely Indefensible | Moderately Indefensible | Somewhat Indefensible | Neither Defensible nor Indefensible | Somewhat Defensible | Moderately Defensible | Completely Defensible |
| --- | --- | --- | --- | --- | --- | --- |
| 1 | 2 | 3 | 4 | 5 | 6 | 7 |

To what extent would you be willing to engage in this behavior?

| Completely Unwilling to Engage in this Behavior | Moderately Unwilling to Engage in this Behavior | Somewhat Unwilling to Engage in this Behavior | Neither Willing nor Unwilling to Engage in this Behavior | Somewhat Willing to Engage in this Behavior | Moderately Willing to Engage in this Behavior | Completely Willing to Engage in this Behavior |
| --- | --- | --- | --- | --- | --- | --- |
| 1 | 2 | 3 | 4 | 5 | 6 | 7 |

QRPs:

1. To enhance chances of publication, violating the ideal of “replace, reduce, refine” regarding the use of research animals.

2. Adding additional research participants because the results collected thus far are not yet statistically significant.

3. Stopping collecting data earlier than planned because the hypothesized result already had been attained.

4. Rounding off a p value simply to make results seem more significant, such as by reporting a p value of .044 to be p = .04.

5. Deciding whether to include or exclude data after looking at the impact of doing so on the results.

6. Failing to report all of a study’s outcome measures.

7. Selectively discussing only studies that supported the hypothesized result(s).

8. Reporting an unexpected result as having been hypothesized from the start.

9. Drawing strong inferences from statistically significant but underpowered results.

10. Selective reporting of subgroups, outcomes, and time points.

11. Deliberately delaying reporting results in order to publish findings in a higher impact journal.

12. Re-use of one’s own previously published ideas or words without citation, such as parts of a literature review section, introduction or methodology, but without re-using data, results, or analysis.

13. Publishing results of a single study as several articles simply to increase the number of publications derived from the research (the so-called “salami slicing” problem).

14. Changing the design, methodology or results of a study to please a sponsor.

15. Acknowledging another’s technical assistance in publication without that person’s permission.

Demographics Questions:

What is your age (in years)? _______

What is your gender?
Male
Female
Other

What is your race?
African American or Black
Asian or Asian American
Caucasian or White
Hispanic or Latino
Other

What is your academic field?

What is the highest degree you hold?

How many years have you been in your field?

How many grants have you earned throughout your career?

How much funding have you acquired throughout your career (in dollars)?

Are you funded by the NIH, NSF, both, or neither?

References:

Sacco, D.F., Bruton, S.V., & Brown, M. (2018). In defense of the questionable: Defining the basis of research scientists’ engagement in questionable research practices. Journal of Empirical Research on Human Research Ethics 13(1). DOI: 10.1177/1556264617743834
